# Mental health in adolescents after experiencing a flood event in Bavaria, Germany—A qualitative interview study

**DOI:** 10.3389/fpubh.2023.1210072

**Published:** 2023-09-06

**Authors:** Alina Schürr, Johanna Elbel, Annika Hieronimi, Isabel Auer, Michaela Coenen, Stephan Böse-O'Reilly

**Affiliations:** ^1^Institute for Medical Information Processing, Biometry, and Epidemiology - IBE, Chair for Public Health and Health Services Research, Medical Faculty, Ludwig-Maximilians University (LMU) Munich, Munich, Germany; ^2^Pettenkofer School of Public Health, Munich, Germany; ^3^Institute and Clinic for Occupational, Social and Environmental Medicine, University Hospital, Ludwig-Maximilians University (LMU) Munich, Munich, Germany

**Keywords:** flood, heavy precipitation, mental health, adolescents, protective factors, stressful factors, extreme weather events

## Abstract

**Background:**

Children and adolescents are particularly vulnerable to the mental health impacts of extreme weather events (EWEs). This qualitative study aims to explore the stressful and protective factors after experiencing an EWE, such as flooding, how adolescents coped with these experiences and what mental health care they received.

**Methods:**

Nine semi-structured interviews were conducted with young adults (18–24 years) living in Simbach am Inn, a German town affected by flooding in 2016. The interviews were analyzed using Kuckartz's qualitative content analysis.

**Results:**

The days after the flood were described as the most stressful time. The main stressors were concern for their family, confrontation with the extent of the damage and uncertainty during the flood. In terms of protective factors, respondents cited talking about the flood, family support and helping with cleanup as the most important. Adolescents requested further mental health care in schools and not just in the immediate aftermath.

**Conclusion:**

Future preventive and therapeutic care measures should be optimized according to protective and stressful factors. Mental health care should be offered after months and should be low-threshold. Additionally, the social environment of adolescents is essential for their mental wellbeing after an EWE and needs to be strengthened.

## 1. Introduction

Due to rising greenhouse gas concentrations and the resulting global warming, the frequency, intensity and severity of extreme weather events (EWEs) are increasing (1, 2). There is no uniform definition, but EWEs are understood as unusual events in a particular place and at a specific time of year, which strongly differ from a reference period (mostly 1961–1990). These include, among others, heatwaves and droughts, storms, avalanches or heavy precipitation with accompanying flash floods (3, 4). According to the World Health Organization (WHO), flooding is among the most common EWEs in Europe and Germany (5). Furthermore, globally, a child born in 2020 will experience, on average, 2.8 times more floods over its lifetime than a person born in 1960, and 1.7 times more if born in Europe under the Paris Agreement commitments (1.5 °*C*-target) (6, 7). At the latest, after the events in Western Europe in 2021, weather extremes such as flooding are no longer a theoretical threat but a danger for a large part of the population in Europe and Germany.

EWEs can have severe health effects, such as injuries or even death (8). But apart from the physical consequences, depending on their severity of the event, flooding can be a traumatic experience and can cause adverse mental health impacts (9–11). Immediately after a flood, acute stress symptoms may occur. As long term mental health consequences, the most common ones are sleep disturbances, concentration problems, or post-traumatic stress symptoms and disorders (9, 12–15).

Children and adolescents are particularly vulnerable to those effects. They are strongly dependent on their caregivers and familiar spatial environments. Moreover, they process traumatic events differently than older people and often lack appropriate coping strategies to deal with difficult situations (9, 16). For this reason it is important to focus on the younger generation in upcoming research regarding the mental effects of EWEs as well as on proper care structures and prevention (1, 2, 9–11, 17, 18).

To date, research has mainly focused on mental health outcomes following natural disasters such as hurricanes. Only a few studies have examined mental health aspects of flooding in children and adolescents, especially in Europe and Germany (19). In addition, there is a lack of research using a qualitative approach to examine the experiences and mental health outcomes of adolescents. Furthermore, most studies focused on both children and adolescents. This paper, however, focuses exclusively on adolescents.

A previous study by Hieronimi et al. interviewed experts like pediatricians and teachers in Simbach am Inn, a small town in Bavaria, Southern Germany to explore the mental health consequences for children and adolescents (20): On June 1st, 2016, flooding occurred in Simbach am Inn [9,736 inhabitants; (21)] after high precipitation. The stream called Simbach overflowed during midday, and a tidal wave rolled toward the town. Large parts of the inner city were devastated. Several streets were underwater, with houses flooded up to the first floor and cars and trucks washed away. The volunteer fire department reported evacuating at least 670 people. In total, five people lost their lives due to the flooding. There were power outages, and the water was turned off for a few days. Additionally, the school was closed for approximately a week afterwards (22, 23). The interviews with experts from Hieronimi et al. showed that the heavy rain event in 2016 affected children and adolescents mentally, and the importance of the social environment for their psychological wellbeing (20). This paper aims to provide an insight into the mental health consequences from the perspective of the young people themselves. This change in perspective is achieved by allowing the young people to speak for themselves.

In conclusion, the aim of this study is to learn from young people who have already experienced such an event 6 years ago in Simbach am Inn (Germany) and to get to know their experiences, the effects on their mental health as well as their demands and needs regarding mental health care with high (e.g., psychotherapy) and low-threshold (e.g., school interventions) services.

These insights are of great interest to public health community to improve patient care in case of existing deficits and provide a basis for further research activities. By exploring protective and stressful factors, adolescents could be helped better in the future following such an EWE.

That leads to the following research questions: (i) What effects on their mental health triggered by the EWE in Simbach 2016 do formerly affected young adults describe? (ii) Which aspects did the affected young people perceive as beneficial (protective factors) or stressful (stressors) for their mental health? (iii) What were young peoples‘ experiences and needs regarding mental health care during and after the flooding?

## 2. Materials and methods

### 2.1. Study design

To explore and explain the situation of young adults during and after the 2016 flood, a qualitative research design using semi-structured interviews was chosen. Qualitative research is for describing peoples' perspectives and to better understand their experiences (24). The participants in this study were between the ages of 18 and 25, so, since the interviews were conducted in 2022 and the flood occurred in 2016, the young adults interviewed experienced the flood as adolescents. Therefore, the risk of re-traumatization is considered low. Nevertheless, a pediatrician was present or attainable during and after the interviews.

### 2.2. Development of the interview guide

The semi-structured interview guide was developed after an in-depth literature review. It was also discussed with an interdisciplinary group of scientists from psychology, public health, and medicine. The existing questions from Karutz et al. were used as guidance (19). The authors developed a partially standardized, semi-structured interview guide for children and adolescents in complex hazardous and injurious situations such as natural disasters (19). The interview guide used in this study contains open questions and is split into four topics: general experiences during and after the flood event, mental health in, mental health care, and reflection of the interview. The first topic of the interview guide contains questions about the general course of events. In the second part, the mental stress of the young adults was asked to gain knowledge about protective and stressful factors. Thereupon, the mental health care during and after the flooding is inquired. In the fourth and last question part, a reflection of the interview and the current emotional state is to be discussed. The interview guide was pilot tested with two young adults to clarify the questions, detect possible errors and check the processing time. The interview guide did not need adaptation during the study process. Included questions of each group are shown in Table 1. Before conducting the interviews, a short questionnaire, together with the invitation, was sent to the participants to get an impression of the educational level, profession and gender.

**Table 1:** Excerpt from the interview guide.

**Topic**	**Leading questions**	**Further questions**
General experiences during and after the flood event	What were you doing on the day of the flood?	How did you hear from the flooding?
	To what extent were you or your social or family environment affected by the flooding?	Was your home affected by the flooding?
Mental health in adolescents; stressors and protective factors	What were your first feelings and thoughts after hearing about the flooding?	What was the first thing you did?
	If you review the time during (the day of the flooding) and also after the flooding (days/weeks/months): At what point did you feel the most stressed?	How did you feel when the current danger was over?
Mental health care	What or who helped you in the situation at that time?	Who could you turn to with your concerns?
	What was the medical and mental health care like at that time?	Have you sought contact with professionals, e.g., crisis intervention team?
Reflection of the interview	What was it like for you to talk about the event?	How are you feeling right now?
	Would you like to get more support, e.g., in the form of another consultation?	Is there anything you can do following the conversation to help you process it?

### 2.3. Ethics approval and consent to participate

Prior to the realization of this study, the ethics committee of the hospital of the University of Munich (LMU) approved the study on September 12, 2022 with project number 22-0668. The data protection authority at LMU hospital also approved the study (project 1691.b). All interviewees participated voluntarily, provided informed written consent and were free to withdraw their participation at any time without disadvantage. The study was conducted in accordance with the Declaration of Helsinki (25).

### 2.4. Participants

The participants needed two characteristics to be included in the study: (i) They had to be between 18 and 25 years old at the time of the interview and (ii) had to be a resident in Simbach am Inn or the surrounding area (50 km) at the time of the heavy rainfall and flooding in June 2016.

Due to the limited number of available study participants, respondents were selected on the convenience sampling method. However, the number of participants was selected according to the principle of data saturation. In this process, interviews are conducted until the participants stop providing new information. This is usually the case after 6–12 interviews (26). In this study, we reached data saturation after conducting nine interviews.

In order to obtain the required number of interviews, the experts interviewed in 2021 in Simbach were contacted by telephone and email. At the same time, Simbach's youth commissioner, various sports clubs and associations such as the local volunteer fire department were contacted. The multipliers asked potential participants whether they were interested in participating in the study and made contact with the study team. Secondly, the study team addressed people in Simbach directly to acquire more people and, additionally, using the snowball-system, participants asked their peer group. Of the 20 young adults contacted, 11 canceled the interview due to time constraints or did not respond to the email invitation for the interview. In addition, the multipliers gave feedback that several people they contacted did not want to talk about their experiences. Therefore, the respondents were nine young adults.

### 2.5. Data collection

One member of the research team (AS; cand. MSc Public Health) conducted semi-structured interviews using the above mentioned interview guide. The interviews were scheduled for October through December 2022 and conducted in person in a confidential setting, e.g., at the respondents' homes. The participants could decide where they wanted to be interviewed. Three interviews took place in a doctor's office. The study physician was available during and after the interviews. The remaining interviews took place at the participants' home or in a confidential place where they worked. Medical support was directly present at three interviews and indirectly present at the other five interviews. During two of the interviews, the study physician was available by telephone at all times and was present shortly after for a follow-up interview, if needed.

The interviews were audio recorded, and postscripts were prepared after each interview to reflect on the interview, briefly describe the interview situation and note specifics. The interviews were conducted in German and relevant citations were translated into English. The interviews lasted from 15 to 35 min with an average of approximately 21 min. The recorded interviews were transcribed literally in MAXQDA according to the transcription criteria of Dresing and Pehl (27) and pseudonymized. Afterwards, the recorded interviews were deleted. The transcripts were not sent to the interviewees for correction. This was done to avoid re-traumatization.

### 2.6. Data analysis

The transcripts were evaluated using qualitative content analysis according to Kuckartz (28, 29). This method is suitable for interpreting qualitative content by systematizing communication by dividing individual text passages into categories. Thus, meaningful aspects are identified and analyzed (30). The data was evaluated using content categories. The development of the categories was mixed deductive-inductive with an iterative process using the strategy of subsumption. Firstly, categories were set a priori based on the literature, the interview guide and the research questions. Secondly, with the research questions in mind, categorizations were developed on the material during primary data analysis. This included creating overarching categories and subcategories. The first draft of the category system was discussed by the research team. After setting the categories, the individual interviews were analyzed and evaluated. Data analysis was performed using the software MAXQDA 2022 (VERBI GmbH, Berlin, Germany). The short questionnaire was analyzed using Excel and added to the transcribed interviews in MAXQDA.

## 3. Results

### 3.1. Interviews

A total of nine young adults were interviewed between October and December 2022. All but one of the interviews were conducted with only one participant. At the time of the interview, the participants were between 18 and 24 years old (21.1 ± 2.5 years), i.e., they were between 12 and 18 years old at the time of the flood (14.9 ± 2.5 years). All participants lived with their parents in or near Simbach am Inn at the time of the event. The group was largely heterogeneous with respect to educational (e.g., secondary school or high school diploma) and occupational status. The majority of participants were female (*n* = 7). Four respondents were directly affected, e.g., they lived in a flooded house or had a flooded basement. Only one participant was affected to the extent that the house was destroyed to the point that it was uninhabitable. Five were not directly affected, but lived in Simbach and the surrounding area (up to 10 km) and either had family members or friends who were directly affected or were involved in acute damage repair.

Some interviewees expressed that it was challenging to talk about what had happened, but none expressed that they felt stressed or burdened afterwards. Furthermore, no one took up the offer of further interviews with the study physician or psychologist.

### 3.2. Main categories

Data from the nine guided interviews were systematically analyzed and compared with respect to the underlying research questions. In this way, relationships between the findings from each case could be established and grouped into overarching categories, which are presented below.

#### 3.2.1. Mental health consequences of the flood event

In the interviews, respondents described short-term stress reactions such as shock, disbelief, and helplessness. Participants reported that they experienced the most mental distress in the days following the actual event. Some described not realizing what had happened at first and seeing the extent of the damage was the most stressful part. One respondent described:

“The next day was the worst. That's when you first saw what it had really done. What was broken.” (p. 05)

Regarding longer-term effects that persist or are still felt months after the flood, interviewees described reactions in thinking and remembering as well as changes in behavior. Participants reported several triggers that made them feel uncomfortable and remember the flood years later. These include the anniversary date, media coverage, such as a book written about the 2016 flood or passing by certain places in Simbach.

In addition, several interviewees reacted differently and with more concern to heavy precipitation than before. One participant described a heavy rain situation as follows:

“We went out to eat [...] so my mom wanted just a little bit of normalcy again. [...] That day it rained hard again and [...] we went home early. So we ate and then we went home because we were afraid that something would happen again.” (p. 03)

Even 6 years after the flood, several participants still struggle with anxiety during heavy rains. Some reported that as soon as the stream gets bigger again, they start to get worried. This leads to changes in their behavior. They start to check the water level of the stream when heavy rains start or keep sandbags at home in case of another flood.

“I don't know why, when you drive by there [the Simbach stream] or at the [river] Inn. But even at the Inn, when you see that it's up, it's just like you look at it again. And when friends pass by and you see it on snapshots or something and then you look at it more closely.” (p. 07)

#### 3.2.2. Stressors

During the interviews, a number of stressors were identified as contributing to a more stressful experience and a delay in the processing of the event: the most common ones were concern for family, uncertainties during the flood, confrontation with the extent of the damage and lack of infrastructure and daily structures. Uncertainties included the extent of the flooding and damage. These affected their concern for their families and were marked by power outages and loss of telephone service.The following statements summarize these stressors:

“That was it, not knowing what will happen now and if everyone is okay.” (p. 02)

Another participant described the feeling as follow:

“The worry, it was kind of all-encompassing. Because nobody knew what was going to be affected.” (p. 04)

Most participants described a sense of relief once they knew their respected family was safe. Furthermore, confronting the extent of the damage was described as a distressing factor.

“It was really unbelievable. It's such a goosebumps moment when you walk into the apartment and everything is broken.” (p. 02)

As a result, some even mentioned that they did not feel able to help with the cleanup. Moreover, many interviewees expressed the lack of infrastructure with power and water outages and the lack of daily structure due to school closures as critical factors in their mental health impact. The unfamiliar situation made them feel more anxious and uncomfortable and also demonstrated the magnitude of the event.

“I think the fact that we didn't have school was also something else, because you were so completely torn out of everyday life and then the structure was missing. And you were kind of completely lost.” (p. 07)

“With the phone network. [...] we were like, ‘Okay, what's going to happen to the infrastructure? I don't know, how are we going to reach anybody?”' (p. 04)

Other factors affecting their mental health included stresses in the social system, e.g., problems at school, end of a relationship, death in the family not related to the flood or seeing their parents worried. Other stressors mentioned were lack of education on EWEs prior to the flood and lack of community flood protection. For a complete list of subcategories related to stressors (see Table 2).

**Table 2:** Overview of stressors and protective factors during and after the flood.

**Stressors**	**Protective factors**
Concerns for close persons (family and friends)	Family as shelter and support
Confrontation with extent of damage	Talking as a coping mechanism
Uncertainties during the flood	Feeling the ability to act
Stresses in social system	Return to everyday life
Lack of infrastructure and daily life structure	Sense of/Support from community
Lack of education on EWEs	Peer group as support
Lack of flood protection from the community (Social) Media as a reminder of the flooding	Technology for communication

#### 3.2.3. Protective factors

Several aspects were identified as helpful and essential in coping with the traumatic event. Family support was the most important protective factor. On the one hand, it provided support and protection for the young adults involved. On the other hand, some of the participants were able to stay with family members, especially grandparents, during the first days or weeks. They recalled that it helped them to be in a protective and functional environment, especially during the water cut-off and not to be exposed to the extent of the damage on a daily basis. One participant recalled:

“We went back to my grandmother's house that day because there was no running water. My mother just said that she didn't want us to come back until there was water.” (p. 03)

In addition, all respondents cited talking as a protective factor and a helpful source for coping after the 2016 flood or even with heavy precipitation in the future. While most had conversations in private settings, such as with family or friends at school, others mentioned teachers or their pastor as important contacts.

“You talked about it [the flooding], you talked about it with MANY people, because of course it was the main topic. And that was not an explicit person, but I think that talking about it a lot (...) was good. I think that was also important, that you processed it somehow.” (p. 02)

“Especially in school. [...] Because I also talked to others and I also talked to some of my classmates or others who were affected by it.” (p. 08)

Another way to deal with the traumatic situation was to help with cleanup or other relief work, such as preparing food for those affected. It was important for the interviewees to remain able to act, and it also helped them to understand what had happened and to contribute to and improve the situation. This was especially important for the indirectly affected respondents.

“I think it [helping with cleanup] helped all of us a lot, and it helped me especially. To understand that [the extent of the flood] and to help somewhere, but also to help the people who were really affected.” (p. 04)

Moreover, returning to daily life as quickly as possible was also beneficial to respondents' mental health. In particular, the appearance of the city and returning to a familiar routine associated with the opening of schools played a decisive role. All the stressful and protective factors mentioned by the respondents are summarized in Table 2.

#### 3.2.4. Mental health care

Regarding mental health care during and after the flood, some were unaware that services were available from relief organizations, psychologists or crisis intervention teams. Others knew about them but did not use them. None of the interviewees expressed a need to talk to a mental health professional or to seek mental health care. Care was described as chaotic and according to the interviewees no one, such as teachers or the fire brigade, knew how to handle the situation well. Respondents recalled, that some schools offered special trips or provided a space to share experiences of the event. For example, one respondent mentioned a walk to a river to reconnect with the element of water with their teacher and to avoid building up fears. Others did not even know if their school offered mental health services regardless of the flood. But despite what was available, participants mentioned that specific mental health services would have been helpful, especially in school and with a time lag. In relation to the stressor of lack of infrastructure, one respondent expressed the following wish:

“I think it would have been helpful [to reopen schools earlier]. Of course, not the day after [the flood]. That would have been impossible, everything was really totally destroyed. But I think it would have been better to go back to school earlier. Where of course there is no obligation to go. I mean, some people deal with it differently and just have no place to live and have to look for a place to stay. But I think it would have been better to have at least a halfway structure.” (p. 07)

## 4. Discussion

This study sheds light on the effects and factors influencing the mental health of young adults who experienced a flood in their youth, as well as their needs in regard to mental health care. The most striking observation was the influence of their families. At the same time, they emerged as not only the most stressful, but also as the most protective factor for the respondents' mental health after the flood.

On the one hand, concern for family and knowledge about their health were described as the most important stressor. On the other hand, family members played a crucial role in terms of support, shelter and conversation partners. As a result, being close to family or removed from the stressful environment were important protective measures that allowed for faster coping and recovery. This also shows that adolescents were highly dependent on their caregivers. These aspects are consistent with previous research (9, 14, 20, 31).

Based on similar findings, Hieronimi et al. concluded that it would be important to provide parents with information on how to interact with their adolescents after an EWE and to train first responders on how to support parents (20). The study referred to existing recommendations for parents by Karutz et al. (19, 20). The results of the interviews with the young adults concerned confirmed once again the importance of implementing these recommended measures. However, attention should especially be paid to affected adolescents without strong family support. The number of loved ones to talk to may be limited, further reducing the critical protective factor of talking. In addition, it may be more difficult to find shelter in the first few days or weeks.

When asked about their initial reaction to the event, respondents primarily described short-term stress reactions. These included shock, helplessness and disbelief. This may have been due to the stressor of lack of education on EWEs and may have been exacerbated by the uncertainties during the flood. After the flood, none of the interviewees recalled seeking or needing professional therapeutic help. However, some expressed a need for an explicit person at school to talk about their experiences and feelings. In addition, it is striking that even today, 6 years after the event, some still have problems with heavy precipitation, even though most of them were only slightly affected. This underlines the need for adapted crisis intervention methods, which is also expressed in the literature (19).

There are several possible approaches to minimizing the stressors and strengthening the protective factors.

Adapted crisis intervention could be to offer classes in school after an EWE. This gives young people the space they need to share their experiences and concerns, with the participation of a professional if necessary. Daniel and Michaela associated supportive counseling with lower mental distress and that it counteracted vulnerability factors (12), supporting this approach. This also resonates with the expressed need for non-compulsory care services after the flood. It could also minimize the stressor of lack of structure in daily life and restore familiar structures as quickly as possible.

In addition, given the uncertainties expressed during the flood, the implementation of educational courses about the possibility of EWEs in their environment and learning how to behave and cope in such situations should be evaluated. Vergunst et al. have suggested that school-based psycho-education programs could be useful in the context of minimizing negative mental health outcomes due to climate change, regardless of EWEs (32).

Another approach to minimizing stressors could be to participate in post-disaster recovery efforts. This could be done at the same time as the aftercare classes mentioned above. As described by some participants, helping with cleanup could help with disbelief, provide structure and a sense of control. This supports previous research. Participation in post-disaster recovery activities was associated with increases in self-esteem and feelings of control (15).

Another adaptation for crisis intervention that participants mentioned was long-term aftercare services, which is also consistent with the literature. According to Kar supportive interventions should be community-based and long-term (14).

Apart from the described approaches, in terms of mental health care, interviewees indicated that emergency response structures such as relief organizations, fire and water rescue services and teachers were not adequately prepared and trained for such an event. Further research with the aforementioned mental health care structures that helped adolescents during this time may provide an important perspective for improving mental health care in the aftermath of flooding. This is in line with the recommendations for structured education programs for volunteer and professional staff of relief organizations from Daniel and Michaela as well as from Kar (12, 14).

In addition, methodological aspects should be discussed, in particular, the sampling. As most of the participants were female, the homogeneity of the gender distribution, could indicate that girls or women may be more open to talking about stressful and emotionally charged topics and less affected by stigma (33). It may also indicate a different perception or willingness to talk about the event or one's own mental health, or an indication of inadequate mental health care. However, some young adults, even regardless of their gender, were not willing to be interviewed, which was reflected in the difficulty of recruitment. This also highlights the method of low-threshold mental health care. Through low-threshold interventions, it would be possible to reach everyone, or at least significantly more young people, regardless of their affection or openness to mental health help.

Therefore, in order to minimize long-term mental health consequences after an EWE and to reduce the mentioned stressors, mental health care after future EWEs should be low-threshold, e.g., in school classes, and should be offered over a longer period of time, e.g., months and years after the flood. With the results of this study, the impact on the mental health of these adolescents could be reduced. This is especially important because they are the adults of tomorrow and should be protected.

### 4.1. Strengths and limitations

This study has several strengths. The findings provide valuable insights into the experiences and mental health challenges of trauma victims following a flood event. In particular, the interviews provided information on protective and stressful factors that may help to tailor future crisis interventions. This study also identified gaps in mental health care that can be addressed with the help of these findings. In addition, it opens up possibilities for further research, as none of the respondents reported feeling burdened or re-traumatized after the interview. Furthermore, this study was conducted according to the COREQ criteria and followed good scientific practice for qualitative research (34).

This interview study also has several limitations. It is susceptible to selection bias in its sampling. Many young adults were unwilling to participate. It is possible that these adults were traumatized and therefore did not want to be interviewed. Furthermore, due to ethical regulations, only people who were of legal age could participate in this study. Therefore, conclusions can only be drawn about adolescents. This in turn means that some perspectives cannot be presented and representativeness is limited due to the qualitative study design.

Given the retrospective design of the study, with the event occurring 6 years ago, another limiting factor is recall bias. Respondents' memories may be distorted and, in retrospect, needs and emotions may have been remembered differently than they actually were. In addition, participants' perceptions are not taken as statements of neutral observers, but as statements of stakeholders. Social desirability plays an important role and may have biased the participants' statements. Stigmatized topics such as mental health are particularly susceptible to this (35). This may also be a reason for difficulties in recruitment. In addition, due to the difference in the length of the interviews, some interviewees' views might be more extensively represented than others.

Mental health care differs between urban and rural areas and between different regions and federal states in Germany. In addition, other EWEs have different levels of damage and are a unique situation with their own dynamics. Furthermore, in Simbach, medical care was not interrupted and professional help was available at all times. Therefore, the results of this study and the conclusions drawn from them may not apply to all locations. In addition, many contextual factors may be relevant to the support and management of a disaster and should be considered. To get a better, broader understanding of how adolescents are affected mentally after experiencing a flood event, further research in different areas of Germany may be needed. In addition, a different approach, e.g., hermeneutic, with the same interview material could provide new insights.

Lastly, other researchers may have coded and extracted information differently due to various perspectives. To increase validation, we implemented several strategies, such as the iterative process of coding and structured discussions among researchers in case of disagreement, to minimize bias during data analysis.

## 5. Conclusions

The risk of further EWEs in Germany is increasing due to climate change. In order to improve mental health care for adolescents after EWEs and to minimize the consequences, care structures need to be more accessible. Schools are particularly suited for this purpose. The importance of the immediate social environment, such as the family, was also evident in this study and needs to be strengthened. Follow-up care should also be provided for at least the first year. Further research should focus on school-based psycho-education programs and training for first responders and caregivers.

## Data availability statement

The data are not available due to data protection regulations in Germany.

## Ethics statement

The studies involving humans were approved by Ethics Committee of the Hospital of the University of Munich (LMU) with the project number 22-0668. The studies were conducted in accordance with the local legislation and institutional requirements. The participants provided their written informed consent to participate in this study. Written informed consent was obtained from the individual(s) for the publication of any potentially identifiable images or data included in this article.

## Author contributions

AS, AH, JE, SB-O'R, and MC: conceptualization. AS and MC: methodology. AS: formal analysis, data curation, visualization, and writing—original draft preparation. AS and IA: investigation. SB-O'R, AH, and MC: writing—review and editing. SB-O'R and MC: supervision. AS and AH: project administration. All authors have read and agreed to the published version of the manuscript.
